# Review on Structures of Pesticide Targets

**DOI:** 10.3390/ijms21197144

**Published:** 2020-09-28

**Authors:** Xiangyang Li, Xueqing Yang, Xiaodong Zheng, Miao Bai, Deyu Hu

**Affiliations:** 1 State Key Laboratory Breeding Base of Green Pesticide and Agricultural Bioengineering, Key Laboratory of Green Pesticide and Agricultural Bioengineering, Ministry of Education, Guizhou University, Guiyang 550025, China; xyli1@gzu.edu.cn; 2 College of Plant Protection, Shenyang Agricultural University, Shenyang 110866, China; sling233@hotmail.com; 3 College of Horticulture, Qingdao Agricultural University, Qingdao 266109, China; zheng.xiao.d@163.com; 4 College of Horticulture, Hunan Agricultural University, Changsha 410128, China; baimiao@hunau.edu.cn

**Keywords:** molecular targets, structures, pesticides, agrochemicals, review

## Abstract

Molecular targets play important roles in agrochemical discovery. Numerous pesticides target the key proteins in pathogens, insect, or plants. Investigating ligand-binding pockets and/or active sites in the proteins’ structures is usually the first step in designing new green pesticides. Thus, molecular target structures are extremely important for the discovery and development of such pesticides. In this manuscript, we present a review of the molecular target structures, including those of antiviral, fungicidal, bactericidal, insecticidal, herbicidal, and plant growth-regulator targets, currently used in agrochemical research. The data will be helpful in pesticide design and the discovery of new green pesticides.

## 1. Introduction

The production of green pesticides is a very complex process, and there are many similarities among the design-synthesis-test-analysis cycles applied in agrochemical research. For example, a product cost of a commercial pesticide is approximately $256 million, and it need screen more than 140,000 compounds and take more than 10 years. Thus, the discovery of a new commercial pesticide faces great challenges [1]. Recently, 862 pesticide types were reported globally. If these commercial pesticide types are classified according to the target of action, there are only 52, 26, and 20 kinds of targets for fungicides, insecticides, and herbicides, respectively [2]. In these targets, nucleic acids synthesis, cytoskeleton and motor protein, respiration, amino acids and protein synthesis, signal transduction, lipid synthesis or transport/membrane integrity or function, sterol biosynthesis, cell wall biosynthesis, melanin synthesis, host plant defense induction are the most used targets for studying the fungicides mechanisms of action [3]. Acetylcholinesterase, *γ*-aminobutyrie acid-gated chloride channel (GABACl), sodium channel, nicotinic acetylcholine receptor (nAChR), glutamate-gated chloride channel (GluCl), juvenile hormone, transient receptor potential vanilloid channel (TRPV), chitin synthase I, insect midgut membranes, mitochondrial ATP synthase, oxidative phosphorylation are the most used targets for studying the insecticides mechanisms of action [4]. Acetyl CoA carboxylase (ACC), acetolactate synthase/acetohydroxy acid synthase (AHAS), microtubule assembly, auxin, D1 serine 264/histidine 215, enolpyruvyl shikimate phosphate synthase, glutamine synthetase, phytoene desaturase, deoxy-D-xyulose phosphate synthase, protoporphyrinogen oxidase (PPO), very long-chain fatty acid synthesis, auxin transport, microtubule organization, hydroxyphenyl pyruvate dioxygenase (HPPD), cellulose synthesis, serine threonine protein phosphatase, solanesyl diphosphate synthase, homogentisate solanesyltransferase, lycopene cyclase are the most used targets for studying the herbicides mechanisms of action [5]. Many pesticides have been discovered that are based on the classical pesticide molecular targets (Table 1). This indicates that the potential new targets of pesticides are very limited, and most pesticides have been developed based on the classical pesticide molecular targets.

**Figure 1:**
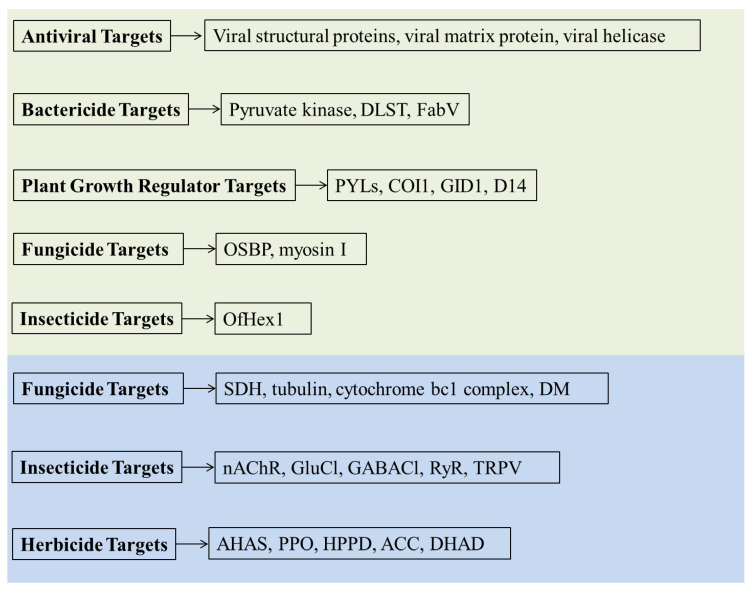
Reviewed the pesticide targets. The potential new molecular targets of pesticides are in light green area; the classical molecular targets of pesticides are in light blue area.

Structural studies of molecular targets have paved a key pathway for understanding the pathogenic mechanism and for the discovery of new green pesticides. Driven by genomics, proteomics, bioinformatics technologies, and chemical biology, many structures of potential molecular targets have been identified owing to the emergence of highly active pesticides. Viral structural proteins, viral matrix protein, viral helicase, pyruvate kinase, dihydrolipoamide S-succinyltransferase (DLST), FabV, PYL family proteins (PYLs), coronatine insensitive 1 (COI1), gibberellin insensitive dwarf1 (GID1), hydrolase DWARF14 (D14), oxysterol-binding protein (OSBP), myosin I, and OfHex1 was applied to developing new green pesticide targets, while succinate dehydrogenase (SDH), tubulin, cytochrome bc1 complex, 14 α-demethylases (DM), nAChR, GluCl, GABACl, ryanodine receptor (RyR), TRPV, AHAS, PPO, HPPD, ACC, and dihydroxy-acid dehydratase (DHAD) are classical pesticide molecular targets (Figure 1). In this review, we summarize the antiviral, fungicide, bactericide, insecticide, herbicide, and plant growth regulator target structures involved in agrochemicals discovery.

## 2. Pesticide Targets

### 2.1. Antiviral Targets

**Figure 2:**
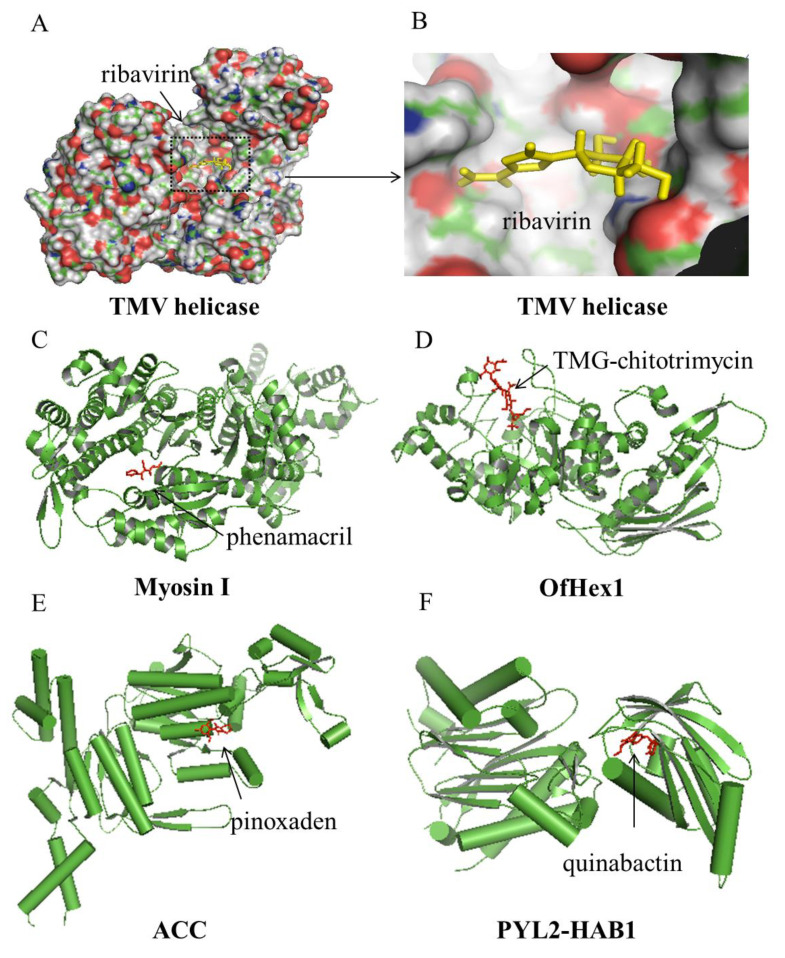
Review of the conformation of the complex structures in agrochemical research. (**A**) Ribavirin binding Tobacco mosaic virus (TMV) helicase, (**B**) Ribavirin in the TMV helicase pocket, (**C**) Phenamacril binding myosin I, (**D**) TMG-chitotrimycin binding Ofhex 1, (**E**) Pinoxaden bimding ACC, (**F**) PYL2-HAB1-quinabactin complex.

Viral coat proteins (CP), virus-like particles, viral helicases, viral matrix proteins, and capping enzymes have been used as targets to develop green anti-plant viral pesticides. The crystal structures of tobacco mosaic virus (TMV) CP [6], cucumber mosaic virus CP [7], potato virus Y virus-like particles [8], tomato mosaic virus helicase [9], rice black-streaked dwarf virus viral matrix protein P9-1 [10], southern rice black-streaked dwarf virus viral matrix protein P9-1 [11], and rice dwarf virus capping enzyme P5 (PDB ID: 5X6Y, unpublished) were solved using X-ray crystallography or cryo-electron microscopy. These structures are regarded as antiviral targets. Based on these structures, the mechanisms of some commercial antiviral agents were studied and revealed. Ningnanmycin breaks down the TMV disassembly by targeting CP [12,13]. Dufulin inhibits the replication of southern rice black-streaked dwarf virus by targeting viral matrix protein at a binding site located inside an internal pore that is stabilized by lateral hydrophobic interactions in the octameric structure [14,15]. An antiviral molecule targeting southern rice black-streaked dwarf virus P10 has been reported [16], and some commercial anti-TMV agents targeting TMV helicase protein have been screened [17,18]. It is noteworthy that ribavirin was screened using a viral helicase with a micro-molar affinity. Further analyses of the structural conformation showed that the target sites of ribavirin were in a shallow groove of the TMV helicase surrounded by D122, S139, D140, K143, and Y274 (indicated by the black arrows in Figure 2A,B [18]. The ribavirin-helicase structure provides a potent complex model for the antiviral discovery.

### 2.2. Fungicidal Targets

Succinate dehydrogenase, tubulin, cytochrome bc1 complex, and 14 α-demethylases (DM) are classical and ideal targets for fungicide discovery. Among these, benzovindiflupyr is a successful succinate dehydrogenase (SDH) inhibitor, the mechanism of benzovindiflupyr is damaging the cell wall, membrane, and organelles, and further inhibits mycelial growth and conidial production of *Bipolaris maydis* [19], and the SDH and 3-nitropropionic acid complex structure were determined in *Gallus gallus*, which can form a covalent adduct of SDH with the side chain of Arg^297^ [20], Tubulin and boscalid complex structure was determined in *G. gallus*, while the tubulin and triazolopyrimidines-complex structure was confirmed in *Bos taurus*, which revealed that triazolopyrimidines are important for complex stability. The result indicated that triazolopyrimidines are microtubule stabilizers targeting the tubulin vinca site [21]. The structures of mitochondrial cytochrome bc1 in complex with famoxadone in *B. Taurus* [22], *Rhodobacter sphaeroides* [23], and *G. gallus* (PDB ID: 3L74, unpublished), were solved, which support an inhibitory mechanism of aromatic–aromatic interaction. The structures of cytochrome bc1 in complex with trifloxystrobin (PDB ID: 3L70, unpublished), azoxystrobin (PDB ID: 3L71, unpublished), triazolone (PDB ID: 3L73, unpublished), and fenamidone (PDB ID: 3L75, unpublished) in *G. gallus* were also solved. The structure of cytochrome bc1 complexed with azoxystrobin in *R. sphaeroides* was solved; the mechanism of azoxystrobin provides a gating mechanism for bifurcated catalyze electron transfer [24]. The discovery of new green fungicides was based on the structures of these mitochondrial cytochrome bc1 complexes [25,26]. The structures of DM complexed independently with S-tebuconazole, R-tebuconazole, S-desthio-prothioconazole, R-desthio-prothioconazole, fluquinconazole, prochloraz, and difenoconazole in *Saccharomyces cerevisiae* were solved, the complex structures reveal triazole-mediated coordination of all compounds and the specific orientation of compounds within the relatively hydrophobic binding site [27], and that of the complex of DM and posaconazole in *Candida albicans* was also confirmed, which provides a molecular mechanism for the potencies of drugs and the intrinsic resistance to fluconazole [28].

Recently, some new fungal targets, such as oxysterol-binding protein (OSBP) and myosin I, were identified. The OSBP-related ligand-binding domain at the C terminus is highly conserved with the specific substrate ergosterol in *S. cerevisiae* [29], with the specific substrate cholesterol in *Kluyveromyces lactis* [30], and with the specific substrate cholesterol in *Homo sapiens* [29,31]. A model of OSBP in *Oomycetes* was built on the basis of published homologous structures, which led to the synthesis and screening of new fungicidal compounds [32]. Myosin I is an important target in *Fusarium graminearum* [33], and the complex crystal structure of phenamacril-bound myosin I in *F. graminearum* was solved. It was discovered that phenamacril binds in the actin-binding cleft of a new allosteric pocket (Figure 2C) [34].

### 2.3. Bactericidal Targets

Compound YZK-C22 inhibits pyruvate kinase by reducing the expression of pyruvate kinase proteins in the metabolic process. Pyruvate kinase is a potential bactericidal target [35] and regarded as a novel target for the discovery of new fungicides [36]. Rice bacterial blight caused by *Xanthomonas oryzae* is the most serious bacterial disease of rice. The potential bactericidal target dihydrolipoamide S-succinyltransferase (DLST) was found using a sulfone compound, which used to confirm the involvement of DLST in the regulation of energy production [37]. The FabV of enoyl-ACP reductase is the key target enzyme in *X. oryzae*. The crystal structure of this protein was solved, and D111, Y236, and K245 were identified as key amino acid residues involved in the inhibition of the reductase activity [38]. This provided important information for the design and synthesis of anti-bacterial blight pesticides.

### 2.4. Insecticidal Targets

At present, the truly commercial and valuable insecticides mainly target nicotinic acetylcholine receptor, glutamate-gated chloride channel, *γ*-aminobutyrate acid receptor, and ryanodine receptor (RyR) [39,40,41,42,43]. Breakthroughs have been made in the development of the insecticides benzamide and chlorantraniliprole, which target RyR, as well as cyclaniliprole. These were discovered based on the allosteric RyR structure [44].

Some new potent insecticide targets have been discovered. The crystal structure of the RyR’s phosphorylation [45] and N-terminal [46] domains, as well as the SPRY2 domain from *Plutella xylostella* [47], were solved. These structures provide insights into the development of novel insecticides [48]. An insect transient receptor potential channel, transient receptor potential vanilloid, is a new and potent molecular target. Afidopyropen was discovered based on the structure of transient receptor potential vanilloid; the role of afidopyropen is a specific modulator of insect TRPV channels [49].

In addition, insect chitinases play crucial roles in chitinous tissues and other physiological processes, and thus are new and potent molecular targets. The crystal structure of insect beta-N-acetyl-D-hexosaminidase OfHex1 [50] and the co-crystal structures with its inhibitors, TMG-chitotriomycin (Figure 2D) [50], PUGNAc [51], and berberine [52], were successfully solved. OfHex1 is an enzyme that linked to an “open-close” mechanism at the entrance of the active site; the active pocket size of OfHex1 to TMG-chitotriomycin was Trp^490^, the active pocket size of OfHex1 to PUGNAc was Val^327^, and the active pocket size of OfHex1 to berberine was Trp^322^, Trp^483^, Val^484^, which contributes to its inhibitory activity. These protein-ligand complexes formed a model for new green insecticide discovery [53,54].

### 2.5. Herbicidal Targets

Acetohydroxyacid synthase (AHAS), protoporphyrinogen oxidase (PPO), and 4-hydroxyphenylpyruvate dioxygenase (HPPD) are widely recognized as the most important herbicidal targets.

For AHAS, the complex structure of its catalyzed subunit with monsulfuron-sulfuron from *Arabidopsis thaliana* was successfully solved, the mechanism of monsulfuron-sulfuron is break the cofactors thiamine diphosphate of AHAS [55], and AHAS was further selected as a potent target for herbicidal discovery [56].

The complex structure of PPO with acifluorfen [57,58] was solved, which shows that the acifluorfen molecule binds to Ile^176^ by forming hydrophobic interactions, and the structural biology of PPO mutants and the mechanism of actions of herbicides based on PPO and its mutants were systematically studied as potent targets of novel herbicides [59,60,61].

In addition, the crystal structures of HPPDs from a variety of different species were systematically studied [62,63,64], and the structures in complex with NTBC [65] and a natural substrate were reported [66]. Thus, the binding mode of the substrate in the enzyme-catalyzed pocket of HPPD was revealed, which laid a solid foundation for an in-depth understanding of the mechanism of action of HPPD-inhibiting herbicides [67,68,69,70].

Some other herbicide targets were reported and utilized as potential molecular targets, such as acetyl CoA carboxylase (ACC) and dihydroxy-acid dehydratase (DHAD). ACC is regarded as a molecular target of phenylpyrazoline herbicide, and the mechanism of pinoxaden acts on ACC (Figure 2E) [71]. The full-length structure of DHAD was solved, and a natural product, aspterric acid, with herbicidal activity targeting the biosynthetic pathway of branched-chain amino acids DHAD was identified [72]. It provides a theoretical basis for designing novel herbicides with new mechanisms.

### 2.6. Plant Growth-Regulator Targets

In the plant growth-regulator target field, the PYL family proteins (PYLs), the jasmonic acid receptor coronatine insensitive 1 (COI1), the gibberellin receptor gibberellin insensitive dwarf1 (GID1), and the strigolactone receptor hydrolase DWARF14 (D14) were new targets.

The PYLs are cellular abscisic acid (ABA) receptors. PYLs, through binding with ABA, undergo conformational changes that result in physical associations and the inhibition of the phosphatase activities of protein phosphatase 2C [73]. Interestingly, PYL2s are the most important molecular targets of plant growth regulators [74]. An X-ray structure of PYL2-quinabactin-HAB1 shows that quinabactin forms a hydrogen bond with the receptor or the protein phosphatase 2C “lock” hydrogen bond network (Figure 2F) [75]. It provides a theoretical basis for designing novel plant growth regulators.

COI1, GID1, and D14 are hormone receptors. A series of receptor structures have been solved. The structure of the complexes formed by COI1 with jasmonate zim domain [76], GID1 with gibberellin [77], D14 with strigolactone [78,79], and decreased apical dominance 2 (DAD2) bound to a quinazolinone derivative [80] were solved, and they could promote the discovery of new plant growth regulators.

## 3. Discussion

**Figure 3:**
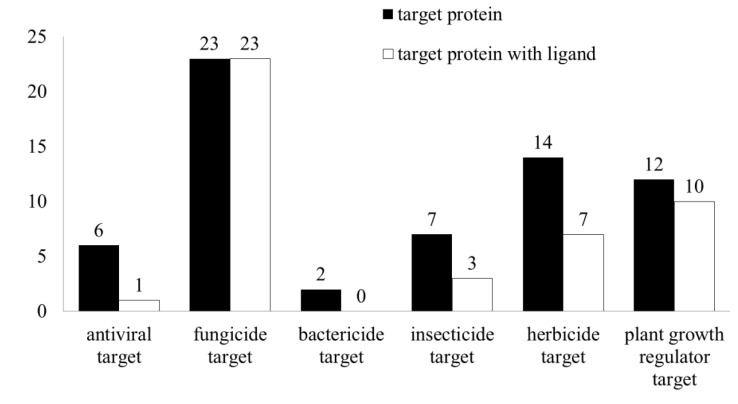
Summary of the number of protein structures in agrochemical research.

Target discovery and validation form one pathway to develop green pesticides. In this review, we summarized 64 potent crystal structures covered in 6 antiviral (Nos. 1–6), 23 fungicidal (Nos. 7–29), 2 bactericidal (Nos. 30 and 31), 7 insecticidal (Nos. 32–38), 14 herbicidal (Nos. 39–52), and 12 plant growth-regulator (Nos. 53–64) target-related agrochemical research studies in the PDB database (Table 2). Among them, 44 crystal structures are those of inhibitors or substrates (Figure 3), and these structural models provide the theoretical basis for discovering new green pesticides.

Among antiviral targets, CP, helicase, matrix, and capping enzyme are the key targets, and the molecular mechanism is to inhibit the viral activity by direct breaking the CP assembly, and/or binding the target site. Among fungicidal and bactericidal targets, SDH, tubulin, cytochrome bc1 complex, DM, DLST, and FabV are the key targets, and the inhibitors play activities by damaging the cell wall, membrane, and organelles, and/or occupying binding site. Among insecticidal targets, nAChR, GluCl, GABACl, RyR, and TRPV are the key targets, and the mechanisms of insecticide usual modulate the conformation of targets to perform the insecticidal effect. Among herbicidal and plant growth-regulator targets, AHAS, PPO, HPPD, ACC, DHAD, PYLs, COI1, GID1, and D14 are the important targets, to study the regulation mechanism of ligand and protein receptors is an important way to discover new pesticides.

With the discovery and development of new green pesticides, many potential molecular targets have emerged. In particular, plant resistance protein, viral CP, and viral minor CP have the most potential for antiviral discovery. For example, harpin binding protein-1 is a potential target activated antiviral response in tobacco by antiviral agent dufulin [81], and tomato chlorosis virus (ToCV), CP play significant roles in sustaining the methyl cycle and S-adenosylmethionine-dependent methyltransferase activity and its minor CP play important roles in silencing suppression activity to counteract the RNA silencing-mediated defense response of the host [82,83]. Glucopyranoside derivatives, pyrimidine derivatives, 4(3H)-quinazolinone derivatives, and novel quinazolinone sulfide inhibitors targets ToCV coat protein with high anti-ToCV activity [84,85,86,87], and its minor coat protein is regarded as a novel target for the new green anti-ToCV inhibitors [88].

With deepening studies of functional genomics, proteomics, computer-aided design, and X-ray crystallography, many new potential molecular targets of pesticides have been identified and structurally characterized. In particular, in plant pathology, the structures of *Phytophthora* effectors PexRD54 and PexRD52, VR3a11 [89,90], and Avh240 [91] were solved, and the complex crystal structures of the *Magnaporthe oryzae* immune receptors RGA5A_S and RGA5A_S–AVR1-CO39 were solved [92,93], which aided in investigating the molecular mechanisms used by the rice disease-resistant protein RGA5 to recognize effectors. These structures provide a basis for studying the mutation-related mechanisms of effectors and provide important data for functional research and fungicide discovery. Interestingly, owing to the molecular dynamics, computational alanine scanning, and site-directed mutagenesis, residue Asn232 in the carboxylesterase gene of *Cydia pomonella* is considered a hot spot for binding with the organophosphate, acephate. Further functional analyses and mutation detection in field populations of *C. pomonella* indicated that the substitution N232A forms a new mutation associated with resistance to organophosphate insecticides in insects [94]. Thus, identifying and utilizing pesticide targets clarifies the molecular mechanisms and toxicity levels of agrochemical compounds at the molecular level. This finding provides important data that can be used to discover new green pesticides having low resistance. With the development of cryo-electron microscopy technology, many target proteins which were difficult to crystallize before can be obtained structures. With the further improvement of resolution, the structures based on cryo-electron microscopy have reached atomic resolution for the first time. This progress makes the interaction between pesticides and targets more accurate, and makes the structure-based drug design easier to realize. The recent interesting founding is the highest-resolution cryo-electron microscopy complex structure of RyR1 and the anthranilic diamide chlorantraniliprole. This complex structure reveals that chlorantraniliprole binds to a pocket on the cytoplasmic side in the voltage sensing domain, and it triggers channel opening and sustained releasing Ca^2+^, and promotes muscle paralysis and achieves insecticidal effect. More interesting, it found that chlorantraniliprole is selective to the diamondback moth over honeybee or mammalian RyRs [95]. These findings provide an important theoretical basis and a foundation for the development of new green pesticides aimed at overcoming resistance.

In the future, pesticide target structures will be at the frontier of agricultural scientific research. (1) Structural analysis is the basis of designing pesticides based on structure. With the progress of computing power and algorithm, under the condition of limited structure, the accuracy of homologous modeling and protein structure prediction is further improved, and the flux of virtual screening is also improved at the geometric level. The screening which was completed in the past few months can be completed in a few weeks or even days, and more and more effective potential pesticides can be obtained, which saves a lot of time and money compared with traditional screening methods. (2) Based on more and more resistant mutations being sequenced, more and more pesticide targets have been found in recent years, and the potential binding sites can be predicted. With the help of the new gene editing technology CRISPR-Cas-9, gene substitution and derivatization can be realized, and it is easier to determine pesticide targets. (3) Machine learning has played a great role in the field of medicine, although this technology is not widely used in the pesticide field. Halicin was developed based on AI, which opened a new door for humans to resist bacterial resistance [96]. We believe that in the near future, AI can also bring a new dawn for the structure-based drug design and development of pesticides.

Pesticide target will be the subject of new technology and innovations in modern agriculture. Target discovery has become the focus of technology and the source of innovation among global agrochemical giants. In 2015, the DuPont Company successfully developed the first fungicide, zorvec, which targets the OSBP and has an excellent control effect on crop diseases with low pesticide resistance. Industry analysts predict that the annual peak sales of zorvec will be $500 million. In China, “Innovative research on new green pesticide and discovery of original target” has been chosen as one of the 60 major technical scientific and engineering problems. Thus, molecular targets will drive the emergence of a number of major new pesticide products, which are crucial for becoming the dominant power in the pesticide market in the future. Molecular target-oriented new green pesticide discovery and development are crucial for stimulating new green pesticide types that are highly efficient, produce low residue levels, and are environmentally safe.

## 4. Conclusions

Small molecule pesticides play specific therapeutic and preventive roles through target-binding to affect the functions of the entire cell or tissue. The safety and effectiveness of green pesticides depend on the functions and differentiation of targets. Our review summarized antiviral, fungicidal, bactericidal, insecticidal, herbicidal, and plant growth-regulator targets in agrochemical research, and pointed out the new potential pesticide targets, including antiviral, bactericidal, and plant growth-regulator targets. It shows that finding pesticide targets that are indispensable for life activities and differentiated among different species is a key scientific goal in the development of selective high-performance pesticides.

## Figures and Tables

**Table 1:** Review of the mechanisms of commercial pesticides.

Pesticide Type	Target Site	Pesticides or Compounds
fungicides	nucleic acids synthesis (e.g., RNA polymerase I and adenosin-deaminase)	phenylamides, hydroxy-(2-amino-) pyrimidines, heteroaromatics and carboxylic acids
	cytoskeleton and motor protein (e.g., *ß*-tubulin)	methyl benzimidazole carbamates, N-phenyl carbamates, benzamides, thiazole carboxamide, phenylureas, benzamides, cyanoacrylates and aryl-phenyl-ketones
	respiration (e.g., complex I: NADH oxido-reductase, complex II: succinate-dehydro-genase, complex III: cytochrome bc1)	pyrimidinamines, succinate-dehydrogenase inhibitors (e.g., phenyl-benzamides, thiazole-carboxamides, and pyrazole-4-carboxamides) and quinone outside/inside inhibitors (e.g., methoxy-acrylates, oximino-acetates, and tetrazolinones)
	amino acids and protein synthesis	anilino-pyrimidines and tetracycline antibiotic
	signal transduction (e.g., MAP/histidine-kinase in osmotic signal transduction)	phenylpyrroles and dicarboximides
	lipid synthesis or transport/membrane integrity or function (e.g., phospholipid biosynthesis and methyltransferase)	phosphoro-thiolates, dithiolanes, heteroaromatics, and oxysterol binding protein homologue inhibitors
	sterol biosynthesis in membranes (e.g., C14-demethylase)	demethylation inhibitors (e.g., piperazines, pyridines, pyrimidines, imidazoles, triazoles, and triazolinthiones)
	cell wall biosynthesis (e.g., chitin synthase and cellulose synthase)	polyoxins and carboxylic acid amides
	melanin synthesis in cell wall (e.g., reductase, dehydratase, polyketide synthase)	melanin biosynthesis inhibitors (e.g., isobenzo-furanone, pyrrolo-quinolinone, triazolobenzo-thiazole, cyclopropane-carboxamide, carboxamide, propionamide, and trifluoroethyl-carbamate)
	host plant defence induction (e.g., salicylate-related, polysaccharide elicitors, anthraquinone elicitors, microbial elicitors, and phosphonates)	benzo-thiadiazole, benzisothiazole, thiadiazole-carboxamide, natural compound (e.g., polysaccharides), plant extract (e.g., anthraquinones, resveratrol), microbial (e.g., bacterial *Bacillus* spp. and fungal *Saccharomyces* spp.), and phosphonates (e.g., ethyl phosphonates)
insecticides	Acetylcholinesterase	carbamates and organophosphates
	*γ*-aminobutyrie acid-gated chloride channel	cyclodiene, organochlorines, and phenylpyrazoles
	sodium channel	pyrethroids, pyrethrins, DDT, and methoxychlor
	nicotinic acetylcholine receptor	neonicotinoids and nicotine
	glutamate-gated chloride channel	avermectins and milbemycins
	Juvenile hormone	juvenile hormone analogues (e.g., hydroprene, kinoprene, and methoprene), fenoxycarb and pyriproxyfen
	chordotonal organ transient receptor potential vanilloid channel	pyridine azomethine derivatives (e.g., pymetrozine and pyrifluquinazon) and pyropenes
	chitin synthase I	clofentezine, diflovidazin, hexythiazox, and etoxazole
	insect midgut membranes	*Bacillus thuringiensis* and *Bacillus sphaericus*
	mitochondrial ATP synthase	diafenthiuron, organotin miticides, propargite, and tetradifon
	oxidative phosphorylation	pyrroles, dinitrophenols, sulfluramid
herbicides	Acetyl CoA carboxylase	Cyclohexanediones, and aryloxphenoxy-propionates
	acetolactate synthase/acetohydroxy acid synthase	triazolopyrimidine, imidazolinone, sulfonylurea, sulfonanilides, and pyrimidinylbenzoates
	microtubule assembly	dinitroanilines, phosphoroamidates, and pyridines
	auxin	phenoxy-carboxylates
	D1 serine 264/histidine 215	triazines, ureas, triazinones, phenylcarbamates, and amides
	enolpyruvyl shikimate phosphate synthase	glyphosate
	glutamine synthetase	phosphinicacids
	phytoene desaturase	phenyl-ethers
	deoxy-D-xyulose phosphate synthase	isoxazolidinones
	protoporphyrinogen oxidase	N-Phenyl-imides and diphenyl ethers
	very long-chain fatty acid synthesis	thiocarbamates, α-chloroacetamides, benzofuranes, and azolyl-carboxamides
	auxin transport	aryl-carboxylates
	microtubule organization	carbamates
	hydroxyphenyl pyruvate dioxygenase	triketones and pyrazoles
	cellulose synthesis	alkylazines and nitriles
	serine threonine protein phosphatase	endothall
	solanesyl diphosphate synthase	aclonifen
	homogentisate solanesyltransferase	solanesyl diphosphate synthase; cyclopyrimorate
	lycopene cyclase	amitrole

**Table 2:** Review of crystal targets with ligands in different species.

No.	Target Protein	Species	Ligand	Target Type	PDB ID	Reference
1	CP	*Tobacco mosaic virus*	no	antiviral target	4GQH	6
2	P9-1	*Southern rice black-streaked dwarf virus*	no		5EFT	unpublished
3	P9-1	*Rice black-streaked dwarf virus*	no		3VJJ	10
4	Helicase	*Tomato mosaic virus*	no		3VKW	9
5	VLP	*Potato virus Y*	no		6HXZ	8
6	P5	*Rice dwarf virus*	S-adenosylmethionine		5X6Y	unpublished
7	SDH	*Gallus gallus*	3-nitropropionic acid	fungicide target	2FBW	20
8	Tubulin	*Bos Taurus*	triazolopyrimidines		5NJH	21
9	DM	*Candida albicans*	S-tebuconazole		5EAB	27
10	DM	*Candida albicans*	R-tebuconazole		5EAC	27
11	DM	*Candida albicans*	S-desthio-prothioconazole		5EAD	27
12	DM	*Candida albicans*	R-desthio-prothioconazole		5EAE	27
13	DM	*Candida albicans*	fluquinconazole		5EAF	27
14	DM	*Candida albicans*	prochloraz		5EAG	27
15	DM	*Candida albicans*	difenoconazole		5EAH	27
16	DM	*Candida albicans*	posaconazole		5FSA	28
17	DM	*Candida albicans*	posaconazole		5TZ1	28
18	bc1 complex	*Bos Taurus*	famoxadone		1L0L	22
19	bc1 complex	*Rhodobacter sphaeroides*	famoxadone		5KKZ	23
20	bc1 complex	*Gallus gallus*	trifloxystrobin		3L70	unpublished
21	bc1 complex	*Gallus gallus*	azoxystrobin		3L71	unpublished
22	bc1 complex	*Gallus gallus*	triazolone		3L73	unpublished
23	bc1 complex	*Gallus gallus*	famoxadone		3L74	unpublished
24	bc1 complex	*Gallus gallus*	fenamidone		3L75	unpublished
25	bc1 complex	*Rhodobacter sphaeroides*	azoxystrobin		6NHH	24
26	Osh4	*Saccharomyces cerevisiae*	ergosterol		1ZHZ	29
27	Osh1	*Kluyveromyces lactis*	Cholesterol		5WVR	30
28	ORP1	*Homo sapiens*	Cholesterol		5ZM5	29
29	Myosin I	*Fusarium graminearum*	Phenamacril		6UI4	34
30	Pyruvate kinase	*Saccharomyces cerevisiae*	no	bactericide target	1A3W	35
31	FabV	*Xanthomonas oryzae*	no		3S8M	38
32	RyR PD	*Plutella xylostella*	no	insecticide target	6J6O	45
33	RyR NTD	*Plutella xylostella*	no		5Y9V	46
34	RyR SPRY2	*diamondback moth*	no		6J6P	47
35	OfHex1	*Ostrinia furnacalis*	no		3NSM	50
36	OfHex1	*Ostrinia furnacalis*	TMG-chitotrimycin		3NSN	50
37	OfHex1	*Ostrinia furnacalis*	PUGNAc		3OZP	51
38	OfHex1	*Ostrinia furnacalis*	berberine		5Y0V	52
39	AHAS	*Arabidopsis thaliana*	monsulfuron-sulfuron	herbicide target	3EA4	55
40	PPO	*Bacillus subtilis*	acifluorfen		3I6D	57
41	PPO	*Homo sapiens*	acifluorfen		3NKS	58
42	HPPD	*Arabidopsis thaliana*	no		1SQD	62
43	HPPD	*Arabidopsis thaliana*	no		1TFZ	62
44	HPPD	*Arabidopsis thaliana*	no		1TG5	62
45	HPPD	*Zea mays*	no		1SP8	63
46	HPPD	*Homo sapiens*	no		3ISQ	unpublished
47	HPPD	*Rattus norvegicus*	no		1SQI	62
48	HPPD	*Pseudomonas fluorescens*	no		1CJX	64
49	HPPD	*Streptomyces avermitilis*	NTBC		1T47	65
50	HPPD	*Arabidopsis thaliana*	HPPA		5XGK	66
51	DHAD	*Arabidopsis thaliana*	aspterric acid		5ZE4	72
52	ACC	*Saccharomyces cerevisiae*	pinoxaden		3PGQ	71
53	PYL10-PP2C	*Arabidopsis thaliana*	ABA	plant growth regulator target	3RT0	73
54	PYL10-PP2C	*Arabidopsis thaliana*	no		3RT2	73
55	PYL2-HAB1	*Arabidopsis thaliana*	ABA		3KDI	75
56	PYL2-HAB1	*Arabidopsis thaliana*	quinabactin		4LA7	75
57	COI1-ASK1	*Arabidopsis thaliana*	incomplete JAZ1 degron		3OGK	76
58	COI1-ASK1	*Arabidopsis thaliana*	JA-isoleucine and the JAZ1 degron		3OGL	76
59	COI1-ASK1	*Arabidopsis thaliana*	JAZ1 degron		3OGM	76
60	GID1	*Oryza sativa Japonica Group*	GA3		3ED1	77
61	GID1	*Oryza sativa Japonica Group*	GA4		3EBL	77
62	DAD2	*Petunia x hybrida*	quinazolinedione		6O5J	80
63	D14-D3-ASK1	*Arabidopsis thaliana*	strigolactone		5HZG	78
64	D3-ASK1	*Arabidopsis thaliana*	no		5HYW	78

## Abbreviations

CP	Coat proteins
TMV	Tobacco mosaic virus
SDH	Succinate dehydrogenase
OSBP	Oxysterol-binding protein
DLST	Dihydrolipoamide S-succinyltransferase
DM	14 *α*-demethylases
GABACl	γ-aminobutyrie acid-gated chloride channel
nAChR	Nicotinic acetylcholine receptor
GluCl	Glutamate-gated chloride channel
TRPV	Transient receptor potential vanilloid channel
RyR	Ryanodine receptor
AHAS	Acetohydroxyacid synthase
PPO	Protoporphyrinogen oxidase
HPPD	4-hydroxyphenylpyruvate dioxygenase
ACC	Acetyl CoA carboxylase
DHAD	Dihydroxy-acid dehydratase
PYLs	PYL family proteins
COI1	Coronatine insensitive 1
GID1	Gibberellin receptor gibberellin insensitive dwarf1
D14	Strigolactone receptor hydrolase DWARF14
DAD2	Decreased apical dominance 2
ABA	Abscisic acid
ToCV	Tomato chlorosis virus
